# Factors Associated with Ivermectin Non-Compliance and Its Potential Role in Sustaining *Onchocerca volvulus* Transmission in the West Region of Cameroon

**DOI:** 10.1371/journal.pntd.0004905

**Published:** 2016-08-16

**Authors:** Laura Senyonjo, Joseph Oye, Didier Bakajika, Benjamin Biholong, Afework Tekle, Daniel Boakye, Elena Schmidt, Elizabeth Elhassan

**Affiliations:** 1 Sightsavers, Haywards Heath, United Kingdom; 2 Sightsavers, Yaoundé, Cameroon; 3 Sightsavers, Accra, Ghana; 4 Ministry of Public Health, Yaoundé, Cameroon; 5 The African Programme for Onchocerciasis Control, Ouagadougou, Burkina Faso; Washington University School of Medicine, UNITED STATES

## Abstract

**Background:**

Community Directed Treatment with ivermectin is the cornerstone of current efforts to eliminate onchocerciasis. However recent studies suggest there are foci where long-term annual distribution of the drug alone has failed to ensure elimination thresholds are reached. It is important to achieve high levels of compliance in order to obtain elimination targets. An epidemiological and entomological evaluation conducted in the western region of Cameroon in 2011 revealed that two health districts remained with a high prevalence of infection, despite long-term distribution of ivermectin since 1996. This paper explores potential factors that may have contributed to the non-interruption of transmission, focusing on ivermectin treatment compliance and the importance of systematic non-compliance within the population.

**Methodology/Principal findings:**

A mixed methods approach was used, including a population-based survey to assess treatment compliance and factors associated and qualitative assessments including focus group discussions and in-depth interviews with key programme stakeholders and drug distributors. Compliance was reported at 71.2% (95%CI: 61.7–79.2%;n = 853/1198). The key factors related to compliance in the most recent round related to either programmatic and delivery issues, primarily absenteeism at the time of the campaign or alternatively individual determinants. An individual’s experience of side effects in the past was strongly associated with non-compliance to ivermectin. Other factors included ethnicity, how long lived in the village and age. There was a high percentage of reported systematic non-compliance at 7.4% (95% CI: 4.3–12.3%; n = 86/1165), higher amongst females. This group may be important in facilitating the sustainment of on-going transmission.

**Conclusions/Significance:**

Efforts to reduce the number of systematic non-compliers and non-compliance in certain groups may be important in ensuring the interruption of transmission in the study area. However, in areas with high pre-control force of transmission, as in these districts, annual distribution with ivermectin, even if sustaining high levels of compliance, may still be inadequate to achieve elimination. Further studies are required to better understand the transmission dynamics and focus of on-going transmission in the study districts.

## Author Summary

Community Directed Treatment with ivermectin is the cornerstone of current efforts to eliminate onchocerciasis. Ivermectin distribution alone has been shown to be able to interrupt transmission but there are foci where long-term distribution of the drug alone has failed to ensure elimination thresholds are reached. Two health districts in the western region of Cameroon remain with high prevalence of infection despite annual distribution of ivermectin since 1996. The study aims to explore factors related to non-compliance in two health districts in the west region of Cameroon. Nearly 30% of the population did not take ivermectin during the most recent round of mass drug administration and there was a significant proportion of the population that had reportedly never taken the drug. The key factors related to drug compliance in the most recent round, related to either programmatic and delivery issues, primarily absenteeism at the time of the campaign, or alternatively individual determinants, such as side effects associated with the drug, ethnicity, age and years lived in the village. Efforts to reduce the number of systematic non-compliers and non-compliance in certain groups are likely to be important in ensuring the interruption of transmission in the study area.

## Introduction

Onchocerciasis is caused by the filarial parasite, *Onchocerca volvulus*, which is transmitted by *Simulium* blackflies. In humans, the adult stage (macrofilariae) are found as worm bundles, within sub-cutaneous nodules or more deeply in the body, living for an average of ten years [1]. The female adult worm produce microfilariae, which are found circulating in the skin and can migrate to the eye [2]. Host immunological responses to infection are responsible for the cutaneous and ocular morbidities, associated with the infection [3].

Ivermectin has been shown to be a safe and potent *O*. *volvulus* microfilaricide [4,5] and has been licensed for the human treatment of onchocerciasis since 1987. Additionally, ivermectin also has an embryostatic effect on the female adult worm, temporarily reducing microfilarial production [4]. Within three to four months following treatment, microfilarial production slowly resumes [5], however, repeated exposure to ivermectin over time, potentially has a cumulative effect on female worm fertility, with the recovery of microfilarial production likely never reaching pre-treatment levels [6,7]. In respect to the longevity of the adult worm, the effect of ivermectin is believed to be limited [7] and therefore any control efforts require long-term, regular distribution of the drug for the entire lifespan of the adult worm. The community-directed treatment with ivermectin (CDTi) approach was therefore developed by the African Programme for Onchocerciasis Control (APOC), to ensure a sustainable model for the routine distribution of ivermectin in the community [8].

APOC was set up in 1995, with the initial aim of control of infection and elimination of onchocerciasis as a public health problem. However, there is growing evidence, especially in meso and lower hyper-endemic settings, that the distribution of ivermectin alone, can lead to the interruption of *O*. *volvulus* transmission in Africa [9,10], with proof of principle in Senegal, Mali [11], Uganda [12], Nigeria [13] and in Sudan [14]. This has led to the recent shift in focus beyond just control of onchocerciasis to the interruption of transmission and the elimination of infection [15].

However, there are also examples of foci where long-term annual ivermectin distribution alone seems to have been inadequate in interrupting transmission [16–19]. A number of models developed to represent *O*. *volvulus* transmission dynamics [20–22] show that various factors may influence the successful elimination; these range from the pre-control endemicity and magnitude of inter-treatment transmission [20], the duration, frequency, timing and coverage of ivermectin [20–22] and individual dynamics and heterogeneous interactions between the vector, parasite and host [23].

One of the key parameters essential for the success of elimination programmes built on preventative chemotherapy (PCT), is the sustained high level compliance amongst the population at risk [24]. Drug coverage is an often reported metric in PCT programmes, however, there is often a disparity between ‘coverage’ which refers to the proportion of eligible people who received drugs, as compared to ‘compliance’ referring to the proportion of eligible people who actually ingested the drugs [25]. Evidence suggests the importance of compliance on residual infection rates [26]. If a significant proportion of the population systematically fail to comply with treatment, then potentially a proportion of the parasite reservoir remains untreated [27]. This may help facilitate transmission and the potential of recrudescence or re-infection amongst those treated, reducing the chances of successful elimination in the foci [28]. The role of systematic non-compliers and the importance of this group has been highlighted in onchocerciasis transmission models [20] but further studies are required in order to determine the infection status of this group and to further explore the impact of systematic non-compliers on progress towards elimination.

Risk factors shown to be associated with treatment coverage and compliance, range from programme and delivery issues to individual recipient characteristics. Programme level factors include issues with the drug delivery or mode of distribution, failure of the community volunteers to distribute the drugs, absenteeism at the time of the campaign or issues of trust in the drug distributors. Individual level factors range from awareness of the campaign or disease, perceived risk from infection and risk or benefit of taking the drug, including the fear of side effects [25,28–33]. Serious adverse events as a result of ivermectin treatment, are a significant risk in some patients co-infected with *Loa Loa* filariasis [34].

Quantifying and understanding drug compliance is extremely important, especially with the recent paradigm shift from control to elimination in onchocerciasis programmes. Therefore, further studies are needed to determine the impact of non-compliance on progress towards elimination.

The study reported here aimed to further explore factors related to non-compliance in two health districts in the west region of Cameroon. An area where ivermectin distribution has been in place since 1996 but the interruption of transmission, as shown in the 2011 epidemiological and entomological evaluation, has not been achieved [18].

## Methods

### Study area

This study focused specifically on Foumbot and Massangam health districts, in the west region of Cameroon. Both districts are rural, with the total population of Foumbot at 93,071 and Massangam at 39,776 (Community-Directed Drug Distributor census 2014). The predominant ethnic group is Bamoun and the two dominant religions are Islam and Christianity. The dominant vegetation cover in the area is that of degraded forest. There are two rainy seasons (March to May and September to October), facilitating black fly reproduction and disease transmission. There are a number of rivers in the area, including the Mbam River, River Nja (in Massangam) and the River Noun (in Foumbot district). The Simulium species found in the area is *Simulium damnosum* complex, specifically *Simulium squamosum A* [35].

The west region of Cameroon has implemented annual ivermectin distribution since 1996. Initial treatment coverage in the region was low (30–35%) but it increased to 60–65% upon the introduction of the CDTi approach in 1999 [National Onchocerciasis Control Programme, personal communication, 11 September 2015]. More recent data from Massangam district, showed a high geographic coverage (number of villages in the district that received ivermectin/total number of villages), whilst the therapeutic coverage (number of persons that received ivermectin/ total population) was more variable, ranging from 67 to 85% (Table 1). In Foumbot district, the geographic coverage was also generally high, remaining at 100% after 2006. Between 2004–2014, the therapeutic coverage, fluctuated between 76% up to 84%.

**Table 1:** Geographic and therapeutic treatment rates in Massangam and Foumbot from 2004 to 2014. (Source: Respective district health officials, 2016).

Massangam District	2004	2005	2006	2007	2008	2009	2010	2011	2012	2013	2014
Therapeutic Coverage (%)	67	77	78	85	83	80	82	81	81	83	81
Geographic Coverage (%)	96	98	100	100	100	100	100	100	100	100	100
**Foumbot District**											
Therapeutic Coverage (%)	76	79	78	80	82	80	84	84	83	84	80
Geographic Coverage (%)	97	98	93	100	100	100	100	100	100	100	100

In Massangam health district, where lymphatic filariasis is supposedly endemic [36], co-administration with albendazole was introduced in 2011. Both districts have a low risk of severe adverse events (SAE) due to loiasis [37] and no SAEs have been reported from these districts since the onset of the mass administration of ivermectin in 1996 (National Onchocerciasis Control Programme, personal communication, 11 September 2015).

An epidemiological and entomological evaluation was conducted in 2011 and found that although the prevalence of microfilariae and nodules were significantly reduced as compared to the 1996 baseline, transmission of *O*. *volvulus* had not been interrupted. In particular, prevalence of infection remained particularly high (microfilaria prevalence over 40%) in select communities in Foumbot and Massangam. Two out of three fly collection sites had infective rates of 0.19% and 0.18% and an annual transmission potential of 70 (Foumbot) and 300 (Massangam) respectively [18].

### Study design

The data collection was carried out in December 2014, three to four months following the last round of community ivermectin distribution, conducted in August and September 2014. The study used a mixed methods approach.

### Population based treatment coverage survey

The survey aimed to verify the reported therapeutic and geographical coverage of ivermectin at the level of the two health districts and understand reasons for non-compliance. The survey followed a two-stage cluster sampling methodology, with the primary cluster (primary sampling unit) the village selected using probability proportional to size. The secondary cluster, the household, was selected using the household listing approach or where household census information was not available, a modified random walk methodology. A questionnaire was administered to everyone normally resident in the household i.e. resident in the last six months, recording key demographic information, if they received and swallowed ivermectin and albendazole where relevant, the reason if they did not, historical ivermectin compliance and information on side effects from taking the drugs.

If anyone was absent at the time of the household visit, the survey team made one return visit later that day. If still not available and if possible, a household member answered on their behalf and this was recorded on the questionnaire. Households that refused to participate were not replaced.

The sample was calculated to estimate a coverage of 80% with 5% precision, at a 95% confidence level. Taking into account a design effect of 4 (based on recommendations from other treatment coverage surveys [38,39]) and non-response of 20%, a total of 1,180 individuals were to be sampled from 236 households across 20 villages.

### Qualitative assessment

Additional operational and programmatic factors, as well as population and social dynamics which may have contributed to sub-optimal compliance, were explored through qualitative methodologies. In five purposefully selected villages focus group discussions (FGDs) were held with the community, two (one male and one female) per village, conducted in French or Pidgin English. These villages were selected based on their availability of historical data on onchocerciasis and to ensure a wide geographical distribution across the two districts. Individuals that took part in the FGDs were included based on having lived in the village for a minimum of 10 years but ideally at least the last 20 years. Key informant interviews were held with both district onchocerciasis focal persons and Community-directed Drug Distributors (CDDs) in the five selected villages. The qualitative assessment was conducted at the same time as the quantitative survey, and was led by social science researchers with experience in conducting qualitative research.

### Ethics statement

The study was approved by the national ethical review committee “Le Comité National d’Ethique de la Recherche pour la Santé Humaine (CNERSH)” in Cameroon.

The study objectives and procedures were explained to all participants in their local languages and written informed consent was obtained from all participants before they were included in the study. Informed assent was provided by minors (under 21 years of age) and caregivers provided consent for their participation.

### Data analysis

All survey questionnaires were double-entered into a pre-designed EpiData 3.0 database which had in built consistency and validation checks. Further consistency, data range and validation checks were also performed in STATA 12.0 (StataCorp. 2011. *Stata Statistical Software*: *Release 12*. College Station, TX: StataCorp LP), the software also used for the analysis.

Descriptive statistics were employed to present simple frequencies of the dependent variables (ivermectin compliance and systematic non-compliance) and its distribution by sex, age, education, occupation, ethnicity, years lived in the village, history of taking the drug, perceived risk from onchocerciasis and self-reported side effects experienced in the past. Chi-squared tests and multivariate logistic regression models were used to assess for the association between various explanatory variables and the dependent variables, participation in the last treatment (ivermectin) round and systematic non-compliance. Age and sex and variables shown to be associated in the univariate analysis with the dependent variable, were included in the multivariate logistic regression model. The likelihood ratio test was used to determine the model of best fit. The dataset was presumed to be self-weighted (based on the probability proportional to size sampling) but the analysis was adjusted for the cluster sampling methodology using robust standard errors based on observed between cluster variability.

For the qualitative component, all interviews and FGDs were audio-recorded and transcribed verbatim. For the analysis, a three stage thematic coding approach was undertaken, using the interview topic guide to help structure the analysis. This was complemented with a more iterative approach which drew on aspects of grounded theory and allowed for new themes and ideas to develop from the interviews and FGDs. NVivo 10 (NVivo qualitative data analysis software, QSR International Pty Ltd. Version 10, 2012) was used to aid the coding and analysis of the transcripts.

Attempts were made to triangulate the data from both the quantitative and qualitative methodologies and to determine where the findings from both results were similar or disparate.

## Results

For the quantitative survey, a total of 1,215 individuals were interviewed from a total of 240 households across 20 villages. A total of 10 CDDs were interviewed and 10 FGDs held, from participants residing in the five purposefully selected villages.

### Study participants

The median age of the survey participants was 27 years old (mean 31 years), with 54.0% of them being female (95% CI: 50.8–57.1%).

Of those aged over 15 years, most had attended formal education, while around a third had not completed primary level (38.1%; 95%CI: 30.6–46.2%). The majority of respondents identified themselves as Muslim (73.6%; 95%CI: 60.5–83.5%) and of Bamoun ethnicity (80.1%; 95%CI: 66.2–89.2%). The primary occupation of the respondents was farming (49.8%; 95%CI: 38.1–61.4%). Most had lived in the village all of their life (69.0%; 95%CI: 61.1–75.9%) and about a quarter (23.5%; 95%CI: 15.4–34.2%) travelled outside the village for periods of two weeks or more.

### Compliance in the last round of MDA

Overall, 71.2% (95% CI: 61.7–79.2%) of participants (all ages), stated they had taken (swallowed) ivermectin during the last distribution. There was no evidence of a difference in compliance between the two districts (p = 0.27). In Massangam district, where ivermectin was co-administered with albendazole, 59.2% (95% CI: 29.1–83.7%) received both drugs.

### Reasons for non-compliance in the last round

A number of reasons for non-compliance were explored in the study, these related to either programmatic/delivery issues (shown in light grey) or individual/community factors (shown in dark grey Table 2).

**Table 2:** Main reasons for not taking ivermectin during last mass drug administration round given during quantitative survey.

Reason	n	%	95%CI
Absent during campaign	112	36.6	25.8–49.0
Fear of side effects	42	13.4	8.5–20.6
Drug distributor did not come	39	12.8	4.7–30.4
Pregnant	33	10.8	6.5–17.4
Did not hear of campaign	24	7.8	4.1–14.4
Reason not given	24	7.8	3.4–16.9
Medicine does not work	18	5.9	0.8–31.6

### Programmatic/delivery issues influencing compliance

Being absent at the time of the MDA delivery was a key factor impacting on non-compliance, reported by a third of those not taking the drug in the last round. This was sometimes related to seasonal migration, with some migrating to the area only for a few months for transhumance or for farming in the fertile plains before travelling to the towns and cities to sell their produce.

Two other key programmatic/delivery issues were the failure of the drug distributor to deliver ivermectin to the household and the lack of awareness by the individual as to the MDA campaign.

The survey data was corroborated by the qualitative findings, which suggested that the inability of participants to access ivermectin was an important barrier, although it was not always clear whether CDDs failed to deliver the drugs to the household or the individuals were just absent at the time of the drug distribution campaign.

“We didn’t receive the Mectizan this year. Some people received the drug … but not everyone”

(FGD participant, 2015).

“Last time I was registered but I didn’t receive it… [ever since I have lived in the village] I have never had it, but then I spend most of my time out of here”

(FGD participant, 2015).

### Individual/community factors influencing compliance

The socio-demographic characteristics independently associated with drug uptake were age, ethnicity and years of residency in the village (Table 3). The lowest compliance was in young adults (aged 20–34 years) at 61.3% (95% CI: 50.4–71.1%). Individuals that identified themselves as Bamileke were nearly three times more likely to take up treatment as compared to the largest ethnic group, the Bamoun (OR = 2.9; 1.8–4.5 p = 0.01). Those who had lived in the village for less than five years were less likely to have taken drugs that those that had lived there for a longer period. Other characteristics, including sex, occupation, education, religion, or perceived personal risk of onchocerciasis showed either weak or no association with ivermectin compliance.

**Table 3:** Adjusted odds ratios for the association of various explanatory variables and ivermectin compliance (multi-variate analysis).

Variable	n	Odds ratio	p-value	95% CI
**Age: Reference, aged 20 to 34 years (n = 261)**				
5–19 years	418	2.2	0.01	1.2–4.0
35–49 years	232	1.6	0.03	1.0–2.6
50–64 years	151	2.5	<0.001	1.6–3.8
65–79 years	78	3.4	<0.001	1.8–6.1
80+	19	1.1	0.84	0.5–2.4
**Sex (n = 552)**				
Female	646	0.7	0.09	0.54–1.1
**Ethnicity: Reference, Bamoun (n = 961)**				
Bamileke	155	2.9	<0.001	1.8–4.5
Tikar	100	0.4	0.14	0.1–1.4
Mbororo	5	0.4	0.001	0.3–0.7
Other	67	1.0	0.91	0.4–2.4
**Side effects: Reference, never had any side effects (n = 1026)**				
In the past (before this round)	98	0.3	0.001	0.1–0.5
**How long lived in village: Reference, greater than 20 years (n = 592)**				
10–20 years	341	0.9	0.81	0.5–1.7
5–9 years	163	0.7	0.32	0.4–1.4
<5 years	88	0.3	0.003	1.1–0.6

Among individual/community level factors, a fear of side effects was the main reason for not taking ivermectin in the last round (Table 2). A total of 166 (14.3%; 11.7–17.4%) individuals (aged five years or over) self-reported they had ever experienced a side effect after taking ivermectin and there was a strong association between reporting side effects in the past and the likelihood of non-compliance in the last round (odds ratio = 0.3; 0.1–0.5, Table 3).

With regards to side effects, the qualitative data corroborated the survey findings indicating that side effects were a major influencing factor on whether or not to take the drugs. The interviews and FGDs highlighted a variety of side effects from ivermectin mentioned by the community, from minor ailments such as swellings and itches to more serious concerns about visual impairment, potential sterility and death.

“When I take it I have a head ache and I feel so cold. It affects my legs and I can’t walk properly, my body swells but after like two days I feel better”

(FGD participant, 2015).

“We have a case here, a woman who got blind and they are saying that it was caused by Mectizan, there is another one who doesn’t walk well, they say it is Mectizan”

(CDD, 2015).

“They exist [people who always refuse the drug]. They say Mectizan kills”

(CDD, 2015).

Additionally some did not take the drugs at the time of the distribution as they were reportedly saved for their alternative benefits, typically to kill hair lice.

“Some …women take the drugs but they don’t swallow, they use it to kill lice on their hair”

(FGD participant, 2015).

Qualitative data suggests that the majority of the local community were aware of onchocerciasis and generally accepted ivermectin to be effective in treating the disease and related morbidities. Only 6% of participants believed the drug was ineffective (Table 2).

“Yes [onchocerciasis is still a problem] though not very much. Before the distribution of the drug, it was more prevalent but it has reduced now”

(FGD participant, 2015).

“There is a great change, people have realized that Mectizan is very important, when you are not feeling fine and swallow the drug [then] you notice a change”

(FGD participant, 2015).

Onchocerciasis was particularly important in Makouopsap village where a number of individuals were known to be blind as a result of infection.

“Yes there are many people suffering from this oncho problem. It is more of a problem here than malaria is”

(CDD, 2015).

The black fly (local name “moute moute”) was also known to be an issue in the studied communities, especially for farmers, who worked in the fertile areas by the side of the river.

“[Black fly biting] is a serious problem… mostly when we are working on the farm, most of our farms are near the rivers”

(FGD participant, 2015).

“If we go to [the] campsite by evening you won’t be able to be in short sleeves because the number of moute moute there is alarming, the campsite is surrounded by three rivers”

(FGD participant, 2015).

### The role of CDDs as a mediating factor in compliance

One issue highlighted in the qualitative part of the study was the role of CDDs in ensuring high quality campaigns and high treatment coverage. More specifically, two aspects of the CDD role were discussed (i) CDDs’ attrition and motivation and (ii) CDDs’ relationship with the community. For example, the district health officials interviewed in the study pointed out that CDDs’ motivations and subsequently attrition rates varied greatly and were undoubtedly reflected in the duration and quality of the MDA campaigns. The CDDs worked primarily as volunteers and their willingness to distribute drugs was driven largely by their attachment to the local community, their religious beliefs and their commitment to good health.

“Even though we were told that we would not be paid, I just said to myself that by helping someone out in this situation, maybe God will someday also help me out in some way. I am happy and motivated to do the work”

(CDD, 2015).

Some CDDs felt that their work was not appreciated by the community, many experienced problems ranging from the community apathy and mistrust around CDDs’ motivations to negative reactions and insults in response to adverse side effects.

“If the community valued me then at least one father or mother would have called me someday and thanked me simply. Just simple gratitude”

(CDD, 2015).

“At first some people were not even taking it because they thought that when they take it is for my own advantage, they thought that the more they take the more I am paid”

(CDD, 2015).

Supervision and support from the health facility workers did not appear to be a strong motivating factor. In fact, a number of CDDs expressed concern over a lack of support they received in handling potential adverse events and they often had to spend their own money to care for the patient with complications.

“I will spend my money and take the person to the hospital and nothing will be done as promised and at the end the family of the concerned will be the one to take [the] charge. I am sure that is the reason why most people now feel discouraged to take Mectizan”

(CDD, 2015).

“I went and gave Mectizan in one compound, I gave it to this child…. The child felt sick and they said it was Mectizan. So the mother of the child came here one night with a machete. Fortunately for me I had travelled to my village. Had it been that [the] child [had] died, I wouldn’t have come back here. So I thought that if this sharing of Mectizan can cause my dead [*sic*] then it is better I resign and allow the indigenes of this place to share it”

(CDD, 2015).

The only positive incentive mentioned by CDDs was training. However, attending training often resulted in financial losses for CDDs, which also discouraged them from work, as one CDD explained:

“They will invite us at Massangam for a meeting we will spend at least 2500frs for transport and when we get there even our transport will not be given back, it’s because of that I resign, I said I cannot be working and spending my money for nothing”

(CDD, 2015).

### Systematic non-compliers

The majority (67.1% (95% CI: 13.6–28.7%)) of survey respondents stated they always took the drugs, while 20.1% (95% CI: 13.6–28.7%) took the drug at least once in the last 5 years. There was however a substantial number of individuals aged at least 5 years and above (7.4% (n = 86; 95% CI: 4.3–12.3%)) who stated they had never taken ivermectin, herein referred to as systematic non-compliers.

Due to the potential of recall bias, individuals were not asked as to the reasons they did not take ivermectin, beyond the most recent round. However, when analysing the reasons given by systematic non-compliers as to why they did not take ivermectin in the last MDA, the most commonly reported reasons were they were absent during the campaign, 36.6% (95% CI:18.7–59.2%) and fear of side effects, 21.8% (95% CI:14.1–32.1%).

The majority (63.1% (95% CI: 53.7–71.6%)) of systematic non-compliers were female (OR = 1.7; 1.1–2.6, p = 0.02). Reasons provided by the female non-compliers included fear of sterility and issues related to irregular menstruation, which they thought may be a result of taking ivermectin.

“Some people don’t take it [ivermectin], especially women…. they have this [pre]conception that Mectizan causes sterility, I have come across people who told me this is the reason they don’t take Mectizan”

(FGD participant, 2015).

“Women are those who mostly refuse. They say they do not know about their menstrual cycle and that it might disturb them”

(FGD participant, 2015).

Being from the Mbororo tribe (nomadics) was strongly associated with systematic non-compliance (p<0.001). Individuals from this group were ten times more likely to state they had never taken the drug as compared to the largest tribe, the Bamoun (OR = 10.9; 6.5–18.0).

## Discussion

Ivermectin has been routinely distributed in Foumbot and Massangam health districts since 1996, however to date there is no evidence of interruption of transmission.

There is still some uncertainty over the relative contribution of various programmatic, epidemiological and ecological factors in sustaining transmission. This study focused on determining the factors associated with drug coverage and compliance, including systematic non-compliance, a potential explanatory factor sustaining on-going transmission.

### MDA compliance

Prior to the introduction of the CDTi approach in the study area, there were some issues in attaining consistently high ivermectin coverage, although more recently reported coverage has been higher. The study, however, showed compliance for the last round of ivermectin distribution was lower (71.2%; 95%CI: 61.7–79.2%) than that reported through the health system reports (80% in Foumbot and 81% in Massangam) and the proportion of systematic non-compliers was high, at 7.4%. Poor compliance and particularly high levels of systematic non-compliance have been highlighted in some earlier research [26,40] as likely contributors to the potential non-interruption of transmission in this area.

### Reasons for non-compliance in the last round

The study suggests that the main reasons for individuals not taking the drugs can be broken down into two key areas, programmatic/delivery issues and individual/community factors.

### Programmatic/delivery issues influencing compliance

Programmatic delivery issues related to low coverage included the absence of individuals on the day of the distribution, often the result of seasonal migration, a common occurrence in the study area. Additionally, there was a noted failure of CDDs to deliver the drug, although it was unclear whether this was a failure on the part of the CDD or was related to individual’s absence at the time of the campaign. Finally, there was an unawareness of the MDA campaign, this could be related to poor sensitization or again linked to the migratory patterns of the population in this area, it is likely interventions to address both issues will need to factored in future campaigns.

### Individual/community factors influencing compliance

With regard to individual/community level factors, the fear of or experience of side effects associated with ivermectin was the main reason for non-compliance despite the fact that the area is at low risk of loiasis related SAEs [37] and no SAEs had been previously reported. The finding is consistent with earlier research [25,41].

Other individual characteristics associated with non-compliance were ethnicity, younger age and shorter residency in the area. With regards to ethnicity, the Bamileke, who had the highest level of compliance in the last round are not indigenous to the study area and it was not clear as to the exact reason for their higher compliance rate, an issue that needs to be further explored in future research. The lowest levels of compliance in those aged 20–35 years is probably related to increased work and mobility amongst this group, while lower compliance among those who had moved into the village in the last five years, may be to do with a lack of awareness of the campaign and/or the risks of onchocerciasis.

Interestingly, there was reference to the topical use of ivermectin added to the hair as a means to kill lice, suggesting that the drug was in some cases valued for its alternative benefits. This highlights the importance of CDDs directly observing the individuals swallowing the drugs to ensure ivermectin is used as intended.

### Role of CDDs as a mediating factor for compliance

Issues of CDDs’ motivation and their relationship with the community came out as a potential mediating factor of compliance in the qualitative part of the study. The findings suggest that poor CDD motivation and mistrust between the community and the CDDs can be related to both a poor quality campaign and poor drug compliance. These data however, comes from the qualitative interviews only and should therefore be treated as a hypothesis, which needs to be tested in future research using quantitative methods.

### Systematic non-compliance

A high proportion of the population reported they never took ivermectin, with strong evidence that women were more likely to be systematic non-compliers, likely related to the fear of side effects related to infertility. The Mbororo tribe were also strongly associated with non-compliance, a group of nomadic pastoralists that migrate and therefore are likely to miss the MDA campaign, unless specific measures are taken to adequately reach this group.

### Study limitations

The study has a number of limitations. First, the original intention of this research had been to investigate prevalence and intensity of infection amongst those individuals that had not taken ivermectin in the most recent round of MDA, to determine if they are a reservoir of infection facilitating on-going transmission. Unfortunately, due to a high number of individuals refusing to have a skin snip biopsy and due to a loss of follow-up of participants that had participated in the coverage survey, not enough data was generated to be able to report reliable findings. Future studies should try and look at this question in more detail, perhaps using a different methodology. A further limitation of the study was the lack of verification of the reported compliance in the study with the CDD records, with only 59.1% of records available at the time of the study. With regards to recall bias, which can be a limitation in coverage surveys, we tried to minimise it by the short period between the treatment round and the survey and by taking a conservative definition of systematic non-compliance, defined here as having never taken ivermectin, which is more likely to be accurately remembered over time.

### Programmatic implications and recommendations

Despite these limitations, the study has important programmatic implications. Future MDA campaigns need to make efforts to increase ivermectin compliance and specifically address the large percentage of systematic non-compliers. Potential interventions include intensifying awareness of the benefits of ivermectin, improving CDDs’ support and motivation and better coordination and timing of MDA.

Consideration needs to be given to seasonal migration patterns and to higher levels of buy-in from tribal leaders, particularly amongst the largest (Bamoun) ethnic group and the Mbororo nomadic pastoralists. The delivery of MDA through the better use of the kinship system, applied for example in Uganda [42], may also help improve compliance amongst various ethnic groups.

Future success of MDA campaigns must also address the community perception and fear of side effects, especially amongst females, potentially ensuring greater involvement of women in the CDTi approach. It is also important to ensure better support to CDDs in educating communities and managing adverse events.

It is important that MDA programmes in the study areas intensify mop up campaigns or potentially move to semi-annual distribution. Finally, epidemiological models suggest that in areas where pre-control force of transmission is high, like in the study area [18], annual ivermectin distribution even with high compliance, may be inadequate to interrupt transmission and achieve elimination by 2025 [43]. It is therefore important that further studies are carried out to better understand the transmission zones in the two health districts and delineate the potential focus of high transmission. With this knowledge, tailored activities can be developed to ensure the maximum chance of interrupting transmission of *O*. *volvulus* in the study area and achieving elimination.

## Supporting Information

S1 Questionnaire (DOCX)

S1 Focus Group Discussion Guide (DOCX)
