# Supplementary material for: Factors Associated with Ivermectin Non-Compliance and Its Potential Role in Sustaining Onchocerca volvulus Transmission in the West Region of Cameroon
Source: PLoS Negl Trop Dis. 2016 Aug 16;10(8):e0004905. doi: 10.1371/journal.pntd.0004905 (PMC4986942; doi:10.1371/journal.pntd.0004905)
Supplement: S1 Questionnaire — (DOCX) [file pntd.0004905.s001.docx]

**Ministry of Health: Onchocerciasis post MDA coverage survey questionnaire** Sheet _ _ / _ _

Month of recent MDA: _ _ / _ _ _ _ Interviewer name: Date of interview: _ _ / _ _ / _ _ _ _

District name: District ID no: Village name: Village ID no:

Name of Household Head: Household ID no:

| Line no | | Name | Age (Years)  If less than 1 year put ‘0’ years and indicate no of months | Sex (1=M, 2=F) | Education level completed  1= Did not go to school; 2= Primary school (did not complete); 3= Primary school (completed); 4= Secondary school (did not complete); 5= Secondary school (completed); 6= tertiary education | Religion  1= Catholic  2= Protestant  3= Muslim  4= other (specify) | Ethnicity or tribe  1= Bamileke; 2= Bamoun; 3=Bangangte; 4=Mbouda; 5= Beti; 6= Other (specify) | Occupation  1= farmer  2= fisherman  3= trader  4= forest worker  5= other (specify)  6= At school  7 = NA | How long have you lived in the village  1= was born here  2= > 20 years  3 = 10-20 years  4= 5-9 years  5= 1-4 years  6 = less 1 year | If you have not lived in the village all of your life, where did you live before here?  Is this an oncho endemic area? | Do you ever travel out of the village for extended periods of time?  1= Yes (specify area and time)  2= No | Do you feel at personal risk from onchocerciasis?  1= Yes, highly  2= Yes, a little  3= No |
| --- | --- | --- | --- | --- | --- | --- | --- | --- | --- | --- | --- | --- |
|  |  |  |  |  |  |  |  |  |  |  |  |  |
|  |  |  |  |  |  |  |  |  |  |  |  |  |
|  |  |  |  |  |  |  |  |  |  |  |  |  |
|  |  |  |  |  |  |  |  |  |  |  |  |  |
|  |  |  |  |  |  |  |  |  |  |  |  |  |
|  |  |  |  |  |  |  |  |  |  |  |  |  |
|  |  |  |  |  |  |  |  |  |  |  |  |  |
|  |  |  |  |  |  |  |  |  |  |  |  |  |

Village ID no: Household head name: Household ID no:

| Line no | | Swallowed the drugs in recent MDA round (show tablets)?  1=Yes, both  2= Yes, ivermectin only  3= Yes, albendazole only  4= No, neither  5= Don’t Know  6= Not eligible | Verification of taken drugs using CDD record  1= Yes, CDD records verify claim  2= No, CDD records do not verify claim  3= No records available | Reason if not taken (one or both)* | Have you taken the same drug (ivermectin) when offered in previous years?  1 = Yes, always  2 = Sometimes but not in the last 5 years  3= Sometimes not every year but at least once in the last 5 years  4 = No, never (or maybe once >10 years ago)  5 = Don’t know | Did you have any side effects from the drugs, this time or in the past?  1= Yes after this round, please specify what side effects  2 = Yes, in the past, specify side effects and year  3 = No | Have you ever had loiasis or a worm travelling across your eye? (Show the picture of the migration of adult worm)  1=Yes; 2 =No; 3= Don’t know | In-depth treatment history and or skin snip taken?  1 = In-depth treatment history taken  2 = Skin snip taken  3= Not applicable |
| --- | --- | --- | --- | --- | --- | --- | --- | --- |
|  |  |  |  |  |  |  |  |  |
|  |  |  |  |  |  |  |  |  |
|  |  |  |  |  |  |  |  |  |
|  |  |  |  |  |  |  |  |  |
|  |  |  |  |  |  |  |  |  |
|  |  |  |  |  |  |  |  |  |
|  |  |  |  |  |  |  |  |  |
|  |  |  |  |  |  |  |  |  |
|  |  |  |  |  |  |  |  |  |
|  |  |  |  |  |  |  |  |  |

***REASON FOR NOT TAKING DRUG** 1= Absent during campaign 2=Did not hear about campaign 3=Drug distributor did not come 4=Pregnant 5=Breast-feeding 6=Underage/too old 7=Fear of side effects 8 =Is healthy 9=Medicine does not work 10= Tired of taking drugs 11=Other (please specify)
