# Supplementary material for: Factors Associated with Ivermectin Non-Compliance and Its Potential Role in Sustaining Onchocerca volvulus Transmission in the West Region of Cameroon
Source: PLoS Negl Trop Dis. 2016 Aug 16;10(8):e0004905. doi: 10.1371/journal.pntd.0004905 (PMC4986942; doi:10.1371/journal.pntd.0004905)
Supplement: S1 Focus Group Discussion Guide — (DOCX) [file pntd.0004905.s002.docx]

**Focus Group Discussion guide**

Preparation notes for Focus Group Discussion (FGD)

The guide below outlines how the FGD will be set up and also outlines the interactive tools (community mapping and historical timeline) that will be used to aid free flowing discussion amongst the participants.

**Facilitators:** 1 facilitator and 1 note taker

**Sampling:** 5 villages will be selected per study area (three in Foumbot and two in Massangam). The villages that have previously been involved in the epidemiological or entomological surveys, will be selected for the qualitative aspects of the study. Additional villages will also be purposefully selected representing a range of treatment coverage, distance from breeding sites and at differing ends of the transmission zone. In areas of on-going transmission, villages will be selected from those sampled for the treatment coverage survey.

Two FGDs will be conducted per village, one all male and one all female

**Participants:** Each FGD will comprise of 8 individuals (over age of 21) that have lived in the village for at least 10 years but ideally the last 20 years. One FGD per village will be conducted with all men and the same for all females. This is to assist in determining differences in risk associated with females and males.

The FGD should be conducted in an area convenient for all participants but an area where ‘onlookers’ will not be encouraged.

**Supplies required:**

- A few large piece of paper, pens and objects from the local community e,g seeds, stones etc
- Paper and pen for note taker
- Audio recorder (minimum 2)
- Ivermectin drug sample
- Picture showing the migration of adult loa loa
- Camera (to photograph potential breeding sites)
- GPS or smart phone with GPS locater downloaded
- Focus Group Discussion Guide
- Informed consent forms
- Ink pad

1. **GENERAL INFORMATION ON THE VILLAGE**

Date of activity (dd/mm/yyyy): _ _ / _ _ / _ _ _ _ Name of facilitator:

Name of note taker:

District name: District ID no:

Name of village: Village ID no:

Name and contact number of village leader:

**Information on participants of focus group discussions**

| Participant number | Name | Age (years) | Sex (M/F) | Approx no. of years lived in the village | Took drugs in recent MDA round? (Y/N) | Informed consent given [add signature] |
| --- | --- | --- | --- | --- | --- | --- |
| 1 |  |  |  |  |  |  |
| 2 |  |  |  |  |  |  |
| 3 |  |  |  |  |  |  |
| 4 |  |  |  |  |  |  |
| 5 |  |  |  |  |  |  |
| 6 |  |  |  |  |  |  |
| 7 |  |  |  |  |  |  |
| 8 |  |  |  |  |  |  |
| 9 |  |  |  |  |  |  |
| 10 |  |  |  |  |  |  |
| 11 |  |  |  |  |  |  |
| 12 |  |  |  |  |  |  |
| 13 |  |  |  |  |  |  |
| 14 |  |  |  |  |  |  |

**Notes for facilitator:** Introduce the purpose of the focus group discussion and the outline of how the activity will run. Participants should be offered the opportunity to decline participating and informed consent should be received from all before proceeding. The FGD should not last more than 2 hours and the participants should be offered refreshments.

1. **COMMUNITY MAPPING**

**Purpose:** To understand the structure of the village and migration of people in and out of the area

**Instructions for Facilitator:** Identify a central point in the village, in order to orientate the group. Ask for a volunteer or if no-one is capable draw the boundaries of the village and any significant internal boundaries. Get someone else to show on the map some key landmarks e,g schools, water source etc. Mark these on the map.

Approximate where the majority of the houses are and highlight where the CDD or VHTs houses are located.

Locate some big towns surrounding the village and mark on the map or approximate with rocks or stones.

**Questions to guide dialogue:**

Can you describe the daily routine of a typical adult male in your community, from the time they get up until they go to bed? I am most interested in where they go within and outside the village. Ask the same for adult females and children [*Ask the community to map the places each group visit on the map*] Probe- what interactions do they have with the river / areas of fast flowing water

(if not mentioned above) What do most people in the community do for work (formal and informal e.g digging in the fields)? Where do they work? [get the community to map out the key locations where they work]

Have most people lived in this community all of their life? [*If no, ask the community to point to households for those who have moved into the area in the last 10 years and the last 5 years.]* Where have they moved from? Why have people moved to the village?

Do many people leave the village for long periods of time? Why do people leave? Probe – do many people leave for seasonal jobs but later return? If so where do they go?

Can you describe any mass migration of people into this area (not just the village) from other areas (temporary or permanent)? If so, where have they come from? When did it occur? Did those people stay?

If not already on the map, ask about any rivers in the area and mark its flow on the map. How far is it from here?

Is the river there all year round or only some times of the year? [*Mark this on the map*]. Probe – Are there tributaries of the river that are only around some times of the year? If seasonal - when do you find them?

Is black fly biting a nuisance in your community [*make sure the community understand what a black fly is and what their bites are like*]? Why do you say this? Which locations do you notice the biting is worst? Which activities do you do where you find you are more likely to being bitten? Has this changed over time?

Where are there any known breeding sites for black flies in this area? Black flies like to breed in fast flowing water, especially where there is white water or rapids, do you have any areas like that near to you? [*Mark on map*] Are they all year around or only occur in the rainy season? [*You will later ask a guide to take you to the potential breeding sites to assess the site and take GPS co-ordinates*]

Has there been any major changes in land use in this area over the time? E.g deforestation. What was the limits of these changes e.g geographical area. When did this occur?**HISTORICAL TIMELINE TOOL**

**Purpose:** To facilitate a community dialogue to understand if and how onchocerciasis has been a problem and how the control initiatives in the community have changed over time.

**Instructions for Facilitator:** Draw a line on a large piece of paper, indicating 5 year intervals, beginning now and depicting the last 20 years. Then ask the group what have been some significant/memorable national events in the last 20 years e.g new president elected or drought. Mark them on the timeline *[if the group is predominantly illiterate use symbols]*. If it is possible get one of the participants to mark the events. Add additional events that are more specific for the village e.g significant deaths in the community. You don’t need too many, they are just meant as a reference for when you discuss events related to Onchocerciasis/River Blindness. Ask the participants to also think of key events in their lives over the last 20 years (do not mark them on the map).

**Questions to guide dialogue**: Try and get a consensus from the group to the following questions.

Onchocerciasis as a problem in the community

Describe how Onchocerciasis (insert local name) is (or isn’t) a problem in this area? What are the symptoms of Onchocerciasis (insert local name)?

Do you have many people who are blind in the community from onchocerciasis? How has the number of blind people changed since (mention significant event on the timeline), were there more or less than now? *[The idea is to get a sense of how blindness has changed over time and to mark this on the timeline as a line/graph]*

Is itchy skin and rashes [*or insert relevant symptoms for Onchocerciasis, although not ocular conditions*] a problem for people in the community? How has the number of people with these conditions changed since (mention few significant events on the timeline), were there more or less than now? *[The idea is to get a sense of how symptoms for Onchocerciasis have changed over time and to mark this on the timeline as a line/graph- in a different colour to the line demarcating those who are blind]* Who in the community is most affected?

Who is at most risk in the community from Onchocerciasis? Why? As this changed over time? Do you feel that you have a personal risk? Why or why not?

MDA for Onchocerciasis

How is onchocerciasis transmitted?

How do you prevent Onchocerciasis (insert local name)? Can it be treated?

Do you receive drugs [*show the drugs*] annually to prevent blindness (or insert local name for Onchocerciasis)? When did you begin to get these drugs [*get a participant to place a stone on the timeline*]? If they no longer get drugs, ask them to also state when they stopped receiving drugs?

Was there any year/s that the community did not receive the drugs? [*Mark on timeline*] Why did you not receive the drugs?

What time of the year do you receive drugs e.g during rainy season, dry season etc? How do you decide when you will receive the drugs? Probe – does the community decide or health officials?

Do you think these drugs are effective in preventing and treating onchocerciasis? Probe to find out the reason why? Does this effect as to whether you wish to take the drugs? Probe for them to explain further

Do most people in the village take these drugs when offered? Why do some take them? Why do some not? Is it convenient for you to take the drugs e.g good time of year?

[*Refer back to the timeline to where it is indicated the drugs were introduced*] – do you think that everyone in the village took the drugs when they were first introduced? How have the percentage of people taking the drugs changed since (mention few significant events on the timeline). Why do you think this changed? [*Mark on the timeline the rough % of those in the community that took the drugs each year or over a 5 years period, in order to get a sense of how uptake has changed over time*]

Are there any people in the community that always miss taking the drugs or refuse to take them? [*try to get information as to their age, sex, occupation, education etc*] Why do they refuse?

Have any of you here ever missed or refused to take the drugs? What happened, why did you miss or refuse?

What problems have you or your neighbours had when taking the drugs? If so what?

Have you or anyone in the community got seriously ill after taking the drugs? Can you describe what happened. Have the number of persons getting ill got more or less since (mention few significant events on the timeline).

Can you describe how drugs for Onchocerciasis (insert local name) are given to you? Who gives the drugs to you? Are they delivered to your house or do you come to a point in the village? How has this changed over time (mention significant events in the community)? How has this impacted on the success of the drug delivery? [*Ask the community to mark the current CDDs houses on the community map and also where drugs are delivered from*]

What information do you hear/sensitization activities are there about the upcoming mass drug administration? What are the key messages? What communication channels do you hear the information through?

Is anyone in the village involved in reviewing or evaluating each round of ivermectin distribution? Please give details of how this happens. Has this led to any changes in the way ivermectin is distributed?Integrated MDA or alternative drug distributions in the community

Are the drugs for Onchocerciasis given to you with anything else? [*Probe – other drugs e.g albendazole for Lymphatic Filariasis, mosquito nets etc*] If yes, ask the community what they think of this? [*Probe – does it make them more or less likely to take the Oncho drugs*?] Have they always been given in combination? [*If no use the timeline to determine when they began taking the drugs together or with something else*]

What other drugs given to you in the community regularly, apart from those not already mentioned? If yes, ask what they are given for and how regularly. Are you more or less likely to take these drugs compared to those given for Onchocerciasis. Why or why not?

Is there anyone in the village with eye worms (loiasis) [*show picture of loa worm migrating across the eye*]? Swollen legs or scrotum (lymphatic filariasis/elephantiasis)? In-growing eyelashes (trachoma/trichiasis)? [*Use local name and probe into how important a problem they are*]

Community Drug Distributors (CDDs)

How many CDDs do you have in your village? How long have the CDDs you have now, been in post? How have the number of CDDs changed since you began to get ivermectin? [Use map to indicate different periods of time – we want to understand the attrition rate of the CDDs]

Do you appreciate the work of your CDDs valued in the community? Why do you say that, can you give specific examples? Can you give examples of how you show your appreciation for the work of CDDs?

Vector control

Has there been any insecticide used in this area to kill black flies? Was this happening before (mention few significant events on the timeline). Has it ever not happened? If it didn’t happen did it make any difference to the number of flies around/ symptoms experienced?

Is there anything else you want to tell me about Onchocerciasis and the treatment of the village with ivermectin?**TOOL 2: In depth interview with Community Drug Distributors**

**Purpose:** To get the CDD/VHTs perspective on MDA for ivermectin and possible reasons as to why it has not been successful in interrupting transmission.

**Interviews:** To be carried out with 1 CDDs/VHTs per village in the same villages as the FGDs. The other CDDs may listen to the interview and add any additional thoughts at the end. The village form needs to be filled in for all villages sampled (both qualitative and quantitative) – the form can be found under Tool 4, treatment coverage survey.

All CDDs names will be recorded on the village information sheet, where each CDD will also be assigned a number, which must be referred to when referring to a CDD in the interview (ie to identify them later)

Background information

Please can you describe your role as a CDD/VHT, specifically your role in distributing ivermectin for Onchocerciasis. What other activities do you cover as a CDD/VHT? [*Probe – distribute other drugs, LLINs, health promotion etc*]

How were you selected to become a CDD/VHT?

What motivated you to be a CDD/VHT? Do you still feel motivated to work as a CDD/VHT? If yes, why and if not why not?

Do you feel valued as a CDD by the community? Why do you say that? By the health workers? Why do you say that?

What remuneration or rewards do you receive from the community or government for your services?

Can you describe how you are supervised during MDA for Onchocerciasis? Who supervises you? Do they come to your community or do you meet somewhere else? What do they cover as part of your superviison? What role does the community play in your supervision? How does that make you feel?

How is onchocerciasis a problem in your community? Has it always been a problem/not a problem? Who is most affected? How does this compare to other health issues in the community e.g malaria.

Can you describe the training you received on the distribution of MDA with ivermectin? Hhow many times did you receive training and when was the most recent? Did you feel it gave you enough information to do your job adequately? What further training would you like?

Do you think the community are aware of the importance of taking the drugs for onchocerciasis?

What sensitization or messages have the community received before the drug distribution? What topics are covered by the sensitization? How often has this happened? How are the messages transmitted e.g CDD, radio, church etc

How do you distribute the drugs in this community? [*Probe – do you visit each household or do the community come to you?*]

Do you carry out a village census prior to each MDA/round of distributing ivermectin?

Is loiasis (eye worm) in this community? Elephantiasis/lymphatic Filariasis? Is trachoma a problem? Are other drugs distributed in the community?

Are the drugs given with other drugs or interventions? Do you think this makes the community more or less likely to take ivermectin? Why or why not?

What is the uptake like amongst the community (for Ivermectin)? How has this changed over time and why?

Do many people refuse to take the drugs, why? Who are they? Do they always refuse or just occasionally? Do they refuse for all drugs or just ivermectin? Why is this the case?

In the last round were many people missing when you distribute the drugs? Who are they and why do they miss? Do you follow up any individuals that do not receive the drugs / do they get a second opportunity to get the drugs?

Do you see each individual take the drugs or do you leave it with them? Do some individuals have difficulties in swallowing the drugs?

What are the main problems you face during each MDA campaign for Onchoceriasis? [*Probe- drug delivery, not enough drugs, reporting, lack of supervision, refusals to take drugs etc*] How could they be overcome?

Are drugs distributed at a good time for the community? Why do you say that?

Do you normally receive enough drugs to cover the whole community? Do you receive the drugs on time? Is there any year when you were not able to distribute the drugs or all of the drugs, why?

Do many people in the community have side effects from the drugs? What do they experience? How many severe adverse reactions in the community have you been aware of, after someone has taken ivermectin? How do you deal with anyone with a severe adverse reaction?

Where do you send your reports to after distributing ivermectin? How long after the distribution is it possible to report? Do you have any difficulties in sending your reports or filling in your reports? If yes, explain further

**TOOL 3: In depth interview with District or MoH Onchocerciasis focal person**

**Purpose:** To get the government health officials perspective on MDA for ivermectin and possible reasons as to why it has not been successful in interrupting transmission.

**Interviews:** To be carried out with the district(s) Onchocerciasis focal person.

**Introductions:** Introduce yourself and the outline of the study and the process of the interview. Ask that it is ok for the interview to be recorded, to help with analysis. Explain that any comments reported if requested can be anonymised at the interviewees request. Go through the informed consent form and obtain their written consent

Questions for district Onchocerciasis focal person

Date of interview: _ _ / _ _ / _ _ _ _ Name of interviewer:

Summary of information on district

|  |  |
| --- | --- |
| Name of (health) district |  |
| Name of Onchocerciasis focal person and mobile number |  |
| Other positions currently held |  |
| Number of years as Oncho focal person |  |
| Total population of district (date & source) |  |
| Ethnicities of population (include rough %) |  |
| Main occupation of population |  |
| Seasonal or perennial transmission?  Months of seasonal transmission |  |
| What months are the rainy season? |  |
| Number of villages |  |
| Number of villages in hyper-endemic area |  |
| Number of villages in meso-endemic area |  |
| Details of main vector and species complex |  |
| Are there any secondary vectors involved in transmission of oncho? |  |
| Are the breeding sites of the vectors well mapped |  |

Questions for in-depth interview with district focal person

How is ivermectin delivered in your district? Has it always been delivered to the communities in the same way ie same delivery platforms? Has the distribution been integrated or co-ordinated delivery with other drugs or interventions e.g LLINs. How has this affected the coverage attained?

Is Onchocerciasis still a problem in your district/foci? Why do you say that? Is it a problem in all areas or certain villages or areas? What makes you say that? Do you have any thoughts as to why these areas have on-going transmission?

What has been the geographical coverage in the area? Are there any specific villages or areas that have been missed or were added later to the MDA schedule? Why were those villages missed?

What has been the programme coverage rates in your district (denominator – population eligible to receive ivermectin)? Are there specific villages or areas that have had consistently low coverage? Do you know why? Was there any action taken to improve coverage?

Has the coverage of other drugs delivered by MDA or LLINs etc been more or less successful than ivermectin MDA? Why do you say that?

Have you always received drugs every year on time? Was there any year that was missed or delayed?

Are the drugs given at the right time of year ie before peak transmission season? Are all the drugs given at the same time in all of the villages?

Have there been many reports of Severe Adverse Events following Onchocerciasis MDA? Have there been any Severe Adverse Events after the community have received other MDAs for other drugs? How have these affected the uptake or coverage achieved for MDA for Onchocerciasis?

Can you describe the community reporting process for drug distributions. Do you receive accurate reports in a timely manner from the CDDs? If not why not? How did this affect your management of MDA?

Can you describe the supervision process for CDDs/VHTs? Do you think it is adequate?

Has there been any mass migration into or out of the area? Do you think this will have altered coverage rates?

Transmission zone

Is there a clear delineation of the transmission zone for Onchocerciasis infection? How sure are you of the edges of the transmission zone? Why do you say that?

Please can you give details of neighbouring transmission zones/districts/areas. Do you think there is cross-over or spill over of infection from these zones [probe – persons, fly migration, closed or open transmission zone etc]? Why do you say that?

[If relevant] Are there any hypo-endemic zones where MDA has or has not been implemented? Do you think this may have contributed to on-going transmission in the foci?

[If relevant] Are there any areas/villages where you think it would be useful to do additional epidemiological or entomological surveys to further define the transmission zone?

Vector control activities

What vector control activities have been on-going? How have these changed over time?

Have you been able to identify all known breeding sites for black flies in the district/foci? How was this done? (get specific details of all sites)? Can you describe the pattern of the breeding sites and the relationship with onchocerciasis transmission in the district population (ie is transmission uniform across the district, or are there areas where you think there is more intense transmission?)

Has there been any changes in land use since ivermectin distribution began? Has there been any significant deforestation that may have affected breeding sites?

Final thoughts

What have been the major successes in the district in relation to Onchocerciasis control?

What have been the issues encountered in the districts [*probe – drug delivery, community refusal to take drugs, human resources, reporting* etc ]

What factors do you think have contributed to the on-going transmission despite many years of MDA?

What do you think needs to be done to interrupt transmission?
